# Adaptive Immunity in Ankylosing Spondylitis: Phenotype and Functional Alterations of T-Cells before and during Infliximab Therapy

**DOI:** 10.1155/2012/808724

**Published:** 2011-09-28

**Authors:** Balázs Szalay, Gergő Mészáros, Áron Cseh, Lilla Ács, Magdolna Deák, László Kovács, Barna Vásárhelyi, Attila Balog

**Affiliations:** ^1^First Department of Pediatrics, Semmelweis University, Bókay János Utca 53-54, 1083 Budapest, Hungary; ^2^Albert Szent-Györgyi Health Center, Department of Rheumatology, University of Szeged, Kálvária sgt. 57, 6725, Szeged, Hungary; ^3^Research Group of Pediatrics and Nephrology, Hungarian Academy of Sciences, Bókay János Utca 53-54, 1083 Budapest, Hungary; ^4^Department of Laboratory Medicine, Semmelweis University, Bókay János Utca 53-54, 1083 Budapest, Hungary

## Abstract

Our aim was to assess the phenotype of T-cell subsets in patients with ankylosing spondylitis (AS), a chronic inflammatory rheumatic disease. In addition, we also tested short-term T-cell activation characteristics. Measurements were done in 13 AS patients before and during the intravenous therapy with anti-TNF agent infliximab (IFX). Flow cytometry was used to determine T-cell subsets in peripheral blood and their intracellular signaling during activation. The prevalence of Th2 and Th17 cells responsible for the regulation of adaptive immunity was higher in AS than in 9 healthy controls. Although IFX therapy improved patients' condition, immune phenotype did not normalize. Cytoplasmic and mitochondrial calcium responses of CD4+ and CD8+ cells to a specific activation were delayed, while NO generation was increased in AS. NO generation normalized sooner upon IFX than calcium response. These results suggest an abnormal immune phenotype with functional disturbances of CD4+ and CD8+ cells in AS.

## 1. Introduction

Ankylosing spondylitis (AS) is a chronic inflammatory rheumatic disease, the best characterized of the diseases belonging to the concept of spondylarthritides. It affects mainly the axial skeleton and the sacroiliac joints [1]. In time, the chronic inflammation of the spine (spondylitis) causes extra bone formation and eventually leads to the fusion of the vertebrae (ankylosis) [2]. The pathogenesis of AS is still unclear, but it is considered to be an autoimmune disease with a strong association with the MHC class I genetic marker HLA-B27 [3]. In any stages of the disease, autoimmune reactions can be associated with peripheral-arthritis, enthesitis, and extra-articular manifestations such as inflammations in the eye, the gastrointestinal tract, and the heart [4], indicating that AS is a systemic immune-mediated disease. This is supported by the number of alterations found in lymphocyte subgroups in peripheral blood.

Specifically, increased numbers of circulating Th2 helper lymphocytes [5] as well as increased numbers of Th17 cells [6] were reported in AS. Regulatory T-cells as major suppressors of the immune system show decreased prevalence, in the blood of AS patients indicating that their lack may contribute to the pathogenesis of the disease [7]. Along the alterations observed in cell prevalence one can assume that T-cell activation properties may also be altered in AS. In rheumatoid arthritis (RA), a further common example of chronic inflammatory arthritides, T-lymphocytes present an increase in intracellular nitric oxide (NO) production along with increased cytoplasmic Ca^2+^ concentrations [8]. This finding raised the notion that some functional alterations of T-lymphocytes were indeed present in autoimmune rheumatic disorders and would contribute to ongoing inflammation risk and progression of the disease. However, to date, no studies have been performed to characterize functional characteristics of short-term T-cell activation in AS.

Effective therapy in AS includes the administration of nonsteroidal antiinflammatory drugs (NSAIDs) and, in unresponsive patients, the use of anti-tumor necrosis factor (TNF)-*α* agents such as infliximab (IFX). IFX is a chimeric anti-TNF antibody that has been shown to be highly effective for the treatment of AS. Although NSAID treatment has only a symptomatic effect and probably does not alter the disease course, IFX targets the specific inflammatory processes of the disease, and thus may potentially influence disease progression [9]. In addition to its action on soluble TNF-*α*, an increasing body of evidence supports the effect of IFX on immune cell prevalence [10–12]. However, no data regarding the impact of IFX on adaptive immune phenotype were reported in AS.

The aim of our comprehensive study was to assess the prevalence of major regulatory cells of adaptive immunity, and also to investigate the short-term T-cell activation characteristics in AS before and during IFX therapy.

## 2. Materials and Methods

### 2.1. Patients

Eleven male and two female patients with active AS meeting the modified New York criteria [13] were enrolled to the study. The age of the patients was (mean ± SD) 43.7 ± 9.2 years, and disease duration was 10.0 ± 5.4 years. Inclusion criteria were (1) Bath ankylosing spondylitis disease activity index (BASDAI) [14] higher than 4, (2) no response to 2 types of NSAIDs and (3) HLA-B27 positivity. Each of the patients was candidate for IFX therapy. The patients are given infliximab at a dose of 5 mg/kg bw intravenously on week 0, 2, and 6 then on every 8th week. Exclusion criteria were any significant comorbidity and the use of any drug excluding NSAID and a proton pump inhibitor. Nine male age-matched healthy volunteers (39.6 ± 6.5 years) served as controls. Written informed consent was obtained from each participant. The work was approved by a local ethical committee, and it was conducted in accordance with the Declaration of Helsinki (1964).

### 2.2. Cell Preparation

24 mL of lithium-heparin anticoagulated blood was taken from all participants. In AS patients, blood samples were taken at 3 distinct time points: just before starting IFX when each of them was on NSAID therapy alone then on Week 2 and 6 after initiation of IFX therapy. Peripheral blood mononuclear cells (PBMCs) were separated with gradient centrifugation using Ficoll-Paque (GE Healthcare Life Sciences, Pittsburgh, PA, USA) and washed twice with Phosphate Buffered Saline pH 7.4 (PBS, Central Pharmacy of Semmelweis University, Budapest, Hungary). 20% of the PBMCs (approximately 5 × 10^6^ cells) was resuspended in PBS and used for cell surface staining, while 80% (2 × 10^7^ cells) was resuspended in modified RPMI (Sigma-Aldrich, St. Louis, Mo, USA) medium (the Ca^2+^ concentration was set to 2 mM) for intracellular measurements.

### 2.3. Surface Staining

To characterize T-cell subset prevalence values, PBMCs were stained with fluorescent antibodies (Becton Dickinson, San Diego, Calif, USA) against cell surface markers according to the manufacturer's instructions. Samples were measured within 1 hour after staining; at least, 300,000 events were recorded for each acquisition.

Cell types were defined as: helper T-cells (CD4+), cytotoxic T-cells (CD8+), Th1 cells (CD4+CXCR3+), Th2 cells (CD4+CCR4+), Th17 cells (CD4+CCR4+CCR6+), regulatory T-cells (Tregs; CD4+CD25+CD127−), naive T-cells (CD4+CD45RA+ and CD8+CD45RA+), and memory/effector T-cells (CD4+CD45RO+ and CD8+CD45RO+). The prevalence of CD4+ and CD8+ cells expressing early and late activation markers (i.e., CD25, CD69, and HLA-DR, resp.) was also determined.

### 2.4. Intracellular Staining

In order to characterize intracellular processes associated with a specific T-cell stimulation, each specimen marked with CD4+ and CD8+ surface markers was loaded with Fluo3AM, Rhod2-AM, Dihydroethidium and DAF-FM diacetate (Molecular Probes, Carlsbad, Calif, USA) sensitive to cytoplasmic Ca^2+^ levels, mitochondrial Ca^2+^ levels, superoxide generation and nitric oxide production, respectively. Staining conditions were identical to those recently reported [15]. The changes in fluorescent signals were monitored up to 10 minutes after the addition of 20 *μ*g/mL in final concentration of phytohaemagglutinin (PHA) (Sigma-Aldrich, St. Louis, Mo, USA), a specific activator of T-cells.

### 2.5. Equipment and Statistical Analysis

All measurements were performed on a BD FACSAria flow cytometer (Becton Dickinson, San Jose, Calif, USA). Cell prevalence values were determined with conventional gating using FACSDiVa software (Becton Dickinson, San Jose, Calif, USA). 

**Figure 1:**
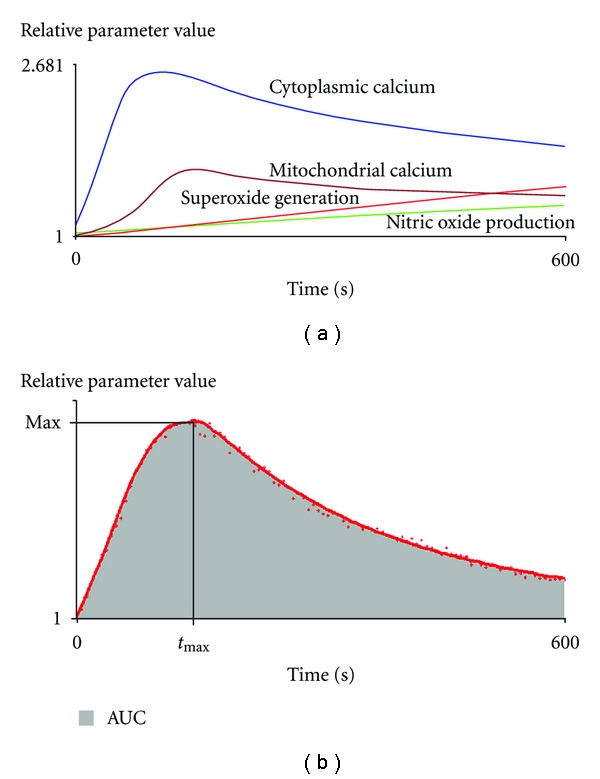
Schematic demonstration of characteristics changes of intracellular processes following PHA activation of lymphocytes. AUC: area under the curve, Max: maximum value, *t*_max _: time to reach maximum.

The kinetic parameters of intracellular processes were determined using R (R Foundation for Statistical Computing, Vienna, Austria) as follows. Measurement timeframe was divided into 100 time intervals of equal length, and the medians of fluorescent values were calculated in each interval. Lowess smoothing method was applied to the median values, and each value was related to that measured at the beginning of the experiment (the resulting values became relative parameter values, rpv). The following parameters were calculated from the rpv values: area under the curve (AUC), maximum value (Max) and time to reach maximum (*t*_max_) (Figure 1). One unit (u) of the AUC value is defined as one rpv in one second. Detailed explanation of these parameters can be found in our previous work [16]. 

Further statistical analysis was based on the values of these parameters and was performed using Statistica 7 software package (Statsoft, Tulsa, Okla, USA). For the comparison of controls and patients before IFX, a Mann-Whitney test was used, while data obtained on Week 2 and 6 were compared to those measured before IFX using paired Wilcoxon test. All data are given as median and [interquartile range]. *P* < 0.05 was considered significant.

## 3. Results and Discussion

Originally, 13 patients were enrolled; on week 2 and 6, 11, and 8 patients provided blood samples, respectively. 2 patients did not return after the initial IFX administration because of technical reasons (moving to a different region), while the others did not attend appointments at the required time.

At the beginning, BASDAI was ≥5 in each AS patient (median [interquartile range]: 6.88 [6.07–7.6]. After 6 weeks of IFX therapy, it decreased significantly: 1.79 [0.60–3.83], *P* < 0.0001.

### 3.1. Cell Prevalence Values

We found several important differences in cell prevalence values between AS with or without IFX and healthy controls (Table 1). The overall prevalence of CD4+ cells within lymphocytes increased in AS, while that of expressing CD25 decreased. In AS, Th1 prevalence values increased by approximately 30 per cent, while Th2 prevalence was double, resulting in a skewness of Th1/Th2 ratio to a Th2 direction. Th17 prevalence increased by 70 per cent, while Treg numbers were comparable to that in controls.

During IFX-treatment, these abnormalities did not disappear. Instead, the prevalence of naive cells on Week 2 and 6 decreased, while the prevalence of memory/effector cells increased on Week 6. 

In general, CD8+ prevalence was comparable in patients and controls irrespectively of IFX therapy.

### 3.2. Functional Characteristics

**Figure 2:**
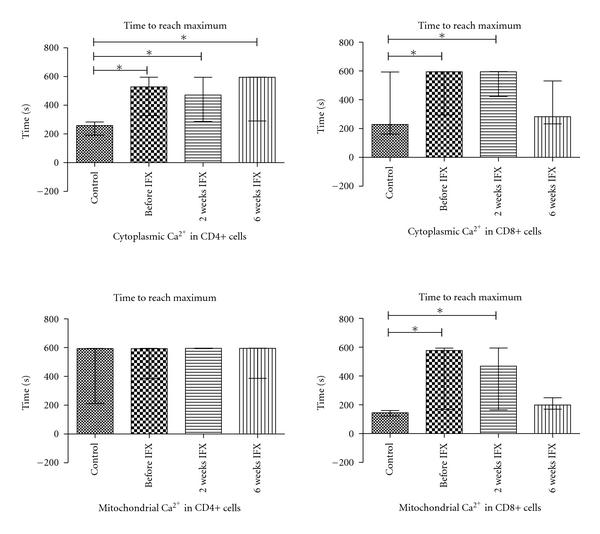
Time to reach maximum in cytoplasmic and mitochondrial Ca^2+^ levels in CD4+ and CD8+ cells following activation in healthy controls (*n* = 9) and ankylosing spondylitis patients before infliximab (IFX) therapy (*n* = 13) then 2 and 6 weeks after initiation of IFX (*n* = 11 and 8, resp.). *P < 0.05.

**Figure 3:**
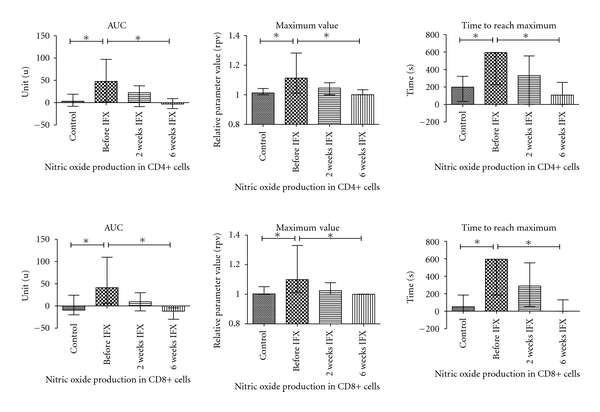
Nitric oxide generation kinetics in CD4+ and CD8+ cells following activation in healthy controls (*n* = 9) and ankylosing spondylitis patients before infliximab (IFX) therapy (*n* = 13) then 2 and 6 weeks after initiation of IFX (*n* = 11 and 8, resp.). *P < 0.05 AUC: area under the curve. For details of IFX therapy, see text.

CD4+ and CD8+ cells presented with a delayed increase in cytoplasmic Ca^2+^ levels after activation in AS compared to controls (Table 2, Figure 2). Mitochondrial Ca^2+^ kinetics also changed in a similar manner in CD8+ cells (Figure 2). The amount of NO generation, max and time to reach max NO levels were significantly higher in CD4+ and CD8+ cells of AS patients (Figure 3). Superoxide generation of T-cells did not differ between AS and controls. (For data on AUC levels see Table 2; additional information on comparable *t*_max_ and Max levels is available upon request.)

With IFX, therapy the delay in CD4+ cytoplasmic Ca^2+^ levels did not normalize in AS. For CD8+ cells, cytoplasmic and mitochondrial Ca^2+^ kinetics during activation normalized by week 6 on IFX (but not on week 2). NO kinetics became comparable in AS patients to that in controls even after 2 weeks of IFX therapy.

### 3.3. Discussion

AS is a chronic, systemic disorder exhibiting autoimmune reactions particularly against large joints and the axial skeleton [9]. The pathogenesis is still unclear, but both innate and adaptive immune responses could have a role in disease development [1].

Different T-lymphocyte subpopulations are implicated in AS [17]. Previous studies extensively investigated the prevalence of different T-cell subtypes in AS in different body fluids. A number of papers consistently reported that peripheral CD4+ [18] and CD8+ [19] cell numbers in blood are increased. In line with these data, we also observed higher than normal CD4+ prevalence in AS patients' blood. 

Simultaneously with increased CD4+, Th1 and Th2 prevalence also increased. This finding supports earlier observations that indicated higher than normal Th2 [5] activity in AS. In addition to CCR4+, the prevalence of CXCR3+ cells were also found to be increased, but to a less extent, leading to a skewness to Th2 direction. Therefore our results relying on cell surface markers reinforce previous data based on the cytokine producing capability of T-cells suggesting a Th2 dominance in AS [20]. (The CXCR3 and CCR4 markers are increasingly accepted as surrogate markers of Th1 and Th2 cells, resp. [12].)

We also tested the prevalence of Th17 and Treg cells due to their central role in the regulation of T-cell subset functions. The clinical importance of Treg cells in autoimmune disorders are underlined by their reduced prevalence in a number of conditions [21], particularly when the prevalence of proinflammatory Th17 cells is increased [22, 23]. For AS, Wu et al. demonstrated that Th17/Treg cell ratios are increased and speculated that this abnormality in immune phenotype may be a contributing factor to disease development [7]. In our patients, we also found a skewness of Th17/Treg ratios to Th17 supporting this theory. 

The discrepancy between the normal Treg prevalence found in our study and the lower Treg reported earlier may be partly due to different techniques used for the assessment of Tregs. While Wu et al. identified Tregs according to FoxP3 expression, we defined Tregs as CD4+CD25+CD127−. Although it is in general accepted that CD127 inversely correlates with FoxP3 in CD4+ cells [24] and that the absence of CD127 expression can be used as an alternative to the transcription factor FoxP3 [25], a recent study suggests that these markers do not necessarily represent the same population of Tregs and, therefore, likely cannot be used for the comparison of results of studies with different methodology [26]. (However, when we analysed the prevalence of cells with CD4+CD25+ positivity—that was used earlier for the detection of Tregs—we did find decreased levels, confirming a decrease in Tregs in AS.)

While CD45RA+ and CD45RO+ (naive and memory/effector) T-cells have an important role in some autoimmune disorders [27], we found no difference in the prevalence values of these cell types between AS and healthy controls confirming the findings of other studies [28]. In addition, the prevalence of T-cells expressing early- and late-onset activation markers (CD69, HLA-DR) was also comparable in AS and controls as in other studies [29]. 

Overall, these results suggest that the prevalence of some CD4+ subsets including Th1, Th2, and Th17 cells is increased suggesting a general alteration in immune phenotype in AS. Of note, no major alterations in CD8+ subsets were found in AS patients. It is worth emphasizing, however, that these results relate to peripheral blood and, therefore, do not necessarily reflect the local conditions in affected joints, where the local immune cell numbers and ratios may be different. 

In this study, we also followed up the cell prevalence values during IFX therapy of AS patients. While during IFX we observed a significant decrease in disease activity, this change was not directly accompanied by the normalization of CD4+ subset prevalence. Instead, the prevalence of naive and memory/effector CD4+ cells significantly altered in an opposite manner leading to an increase in the ratio of memory/naive cells. As the prevalence of cell types investigated did not normalize during IFX, it is reasonable to postulate that the prevalence of these T-cell subtypes are not linked to clinical efficacy of IFX in AS.

These results are in contrast with the effects of IFX on immune cells in other diseases. An increase in the prevalence of CD4+ and CD8+ cells in Crohn's disease [10], that of Tregs [11] and Th1 committed T-cells in RA [12], was observed during IFX treatments. The discrepancy between our results and other studies may suggest that the impact of IFX on cell prevalence may differ between disorders.

Increased chronic TNF-*α* exposure is the hallmark for many inflammatory rheumatological disorders. In AS, high TNF-*α* levels have consistently been reported [30, 31]. High TNF-*α* induces systemic and local inflammation leading to the clinical signs and symptoms of AS. More recently, an increasing amount of evidence has suggested the impact of TNF-*α* on intracellular T-cell signaling [32, 33]. 

TCR stimulation induces Ca^2+^ influx and, through inositol-1,4,5-triphosphate (IP3), the release of Ca^2+^ from intracellular stores. (Almost immediately, the reuptake of Ca^2+^ to endoplasmic reticulum and mitochondria is also initiated.) This process was clearly detected in CD4+ and CD8+ lymphocytes in our experiments: after a specific stimulation of TCR with PHA cytoplasmic Ca^2+^ levels and, in a parallel manner, mitochondrial Ca^2+^ levels increased in CD4+ and CD8+ cells in each sample. Of note, the increase of cytoplasmic Ca^2+^ levels in CD4+ and CD8+ cells from AS is delayed compared to controls. As a consequence, mitochondria (at least in CD8+ cells) also presented a delayed calcium response during short-term activation. (For CD4+ cells, the time when max calcium values in mitochondria reached was beyond the measurement in a large number of control and AS samples.) The delayed calcium response of AS fits well into the observations done in *in vitro* tests with T-cells exposed to TNF-*α*. Church et al. reported that TNF-*α* suppressed the Ca^2+^ peak after PHA stimulation; they have suggested that either signalling pathways upstream of Ca^2+^ mobilisation or the Ca^2+^ signalling itself were impaired by prolonged TNF-*α* exposure [33]. In AS, only one study has been performed with a more robust analytical approach: Lee et al. did not observe a significant difference in intracellular Ca^2+^ between AS patients and normal controls in activated peripheral blood mononuclear cells [34]. The inconsistency between their and our results is probably due to different methodology and cell types investigated.

The increase in Ca^2+^ levels following TCR stimulation triggers a large number of intracellular events. These include elevated NO production [35]. There is also a negative feedback, as high NO levels inhibit TCR signaling by decreasing the zeta-chain expression [36]. The effect of TNF-*α* on intracellular NO production, however, is rather independent on TCR as it induces iNOS activity directly in macrophages [37]. Therefore, in the presence of high TNF-*α* levels, in addition to the decrease of calcium response in T-cells, one can assume an increase in NO levels upon stimulation. This theory is clearly supported by our measurements indicating high NO levels in AS patients.

IFX is an effective anti-TNF agent that suspends TNF-mediated effects. In the prospective phase of our study, cell calcium handling (i.e, cytoplasmic and mitochondrial calcium response upon activation) of CD8+ cells normalized on week 6 (but not on week 2) of IFX therapy. For CD4+ cells, calcium handling remained abnormal even in the presence of significant improvement of clinical status of AS patients on IFX therapy. On the other hand, intracellular NO metabolism became normal already on week 2 and remained comparable to controls on week 6 of IFX therapy both in CD4+ and CD8+ cells. Therefore, one can conclude that the impact of IFX therapy on calcium handling and NO metabolism of T-cells differs. As NO kinetics in activated T-cells normalizes much earlier than calcium-kinetics, the major factors leading to NO abnormalities in IFX untreated patients seem independent of those leading to abnormalities in calcium kinetics. In addition, as the clinical condition of AS patients improved irrespectively of calcium kinetics, it is also reasonable to postulate that Ca^2+^ handling abnormalities of stimulated T-cells do not have a dominant role in the development of clinical signs and symptoms in AS.

In addition to Ca^2+^ and NO, we also tested superoxide generation of activated T-cells. No difference was observed between control and AS patients with or without IFX.

## 4. Conclusions

In this study, we found that the prevalence of Th2 and Th17 cells responsible for the regulation of adaptive immunity is different between AS and healthy controls. While IFX therapy improved the overall condition of patients, cell prevalence abnormalities did not disappear.

Some intracellular processes that are integral parts of lymphocyte activation are also altered in AS. These include intracellular nitric oxide and calcium handling. There are marked differences in the impact of anti-TNF therapy on these processes.

## Figures and Tables

**Table 1:** Prevalence and ratios of T-cell subsets in ankylosing spondylitis patients before and during infliximab (IFX) therapy.

Members of adaptive immunity	Healthy controls (*n* = 9)	Before IFX therapy (*n* = 13)	Week 2 after IFX therapy (*n* = 11)	Week 6 after IFX therapy (*n* = 8)
CD4+	35.80 [29.43-40.95]	42.7 [38.35–48.65]*	47.10 [43.00–49.10]*	44.30 [42.15–48.85]*
CD4+CD45RA+	49.50 [40.68–61.68]	50.80 [45.20–65.95]	50.90 [36.20–55.40]^#^	42.55 [39.55–60.78]^#^
CD4+CD25+	7.55 [6.20–7.88]	4.82 [4.22–5.42]*	3.92 [3.60–4.85]*	4.35 [4.04–4.98]*
CD4+CD69+	3.23 [2.83–4.48]	3.05 [2.72–3.28]	3.04 [2.69–3.43]	2.88 [2.21–3.36]
CD4+HLA-DR+	2.8 [2.53–3.81]	3.03 [2.09–3.46]	1.95 [1.56–2.33]	2.89 [2.21–3.51]
CD4+CD45RO+	42.95 [33.33–49.65]	41.50 [29.40–49.55]	45.50 [37.80–54.60]	47.45 [33.10–54.78]^#^

Th1	9.81 [8.95–12.53]	12.90 [11.75–13.80]*	14.00 [11.50–15.70]*	13.80 [12.43–18.83]*
Th2	4.54 [4.19–4.84]	9.18 [7.32–11.45]*	8.70 [7.98–11.30]*	10.90 [8.32–12.50]*
Th1/Th2 ratio	2.38 [1.95–2.64]	1.31 [1.06–1.85]*	1.40 [1.16–1.83]*	1.38 [1.11–1.72]*
Th17	0.69 [0.60–0.77]	1.18 [1.02–1.69]*	1.2 [0.84–1.55]*	1.26 [0.91–1.51]*
Treg	4.42 [3.76–5.58]	4.45 [3.57–5.51]	4.29 [3.66–5.72]	4.42 [4.05–5.24]
Th17/Treg ratio	0.14 [0.13–0.21]	0.27 [0.19–0.45]*	0.25 [0.17–0.39]*	0.29 [0.18–0.37]*

CD8+	18.00 [14.35–27.98]	17.70 [14.65–21.45]	18.30 [14.20–21.90]	16.65 [14.43–21.60]
CD8+CD45RA+	62.85 [53.38–75.78]	70.30 [61.70–78.75]	69.10 [55.20–72.50]	60.20 [54.43–70.33]
CD8+CD25+	2.84 [1.87–4.06]	2.81 [2.23–3.63]	2.85 [2.07–3.23]	3.29 [1.84–3.54]
CD8+CD69+	4.0 [3.53–12.78]	4.88 [3.37–5.89]	3.71 [3.26–6.77]	5.06 [3.55–6.27]
CD8+HLA-DR+	3.58 [3.10–5.31]	3.43 [2.95–4.33]	4.37 [2.93–5.15]	3.57 [2.91–5.15]
CD8+CD45RO+	23.65 [15.90–28.15]	19.40 [14.25–28.20]	26.30 [19.10–34.90]	28.45 [18.53–40.20]
CD4/CD8 ratio	2.07 [1.32–2.83]	2.26 [2.02–3.15]	2.61 [1.99–3.21]	2.82 [1.90–3.36]

Data are expressed as median [interquartile range] *versus control *P* < 0.05; ^#^versus before IFX *P* < 0.05.

**Table 2:** Functional characteristics of intracellular processes in CD4+ and CD8+ cells following PHA activation in ankylosing spondylitis patients before and during infliximab (IFX) therapy.

	Parameter	Healthy controls (*n* = 9)	Before IFX therapy (*n* = 13)	Week 2 after IFX therapy (*n* = 11)	Week 6 after IFX therapy (*n* = 8)
Cytoplasmic Ca^2+^					

CD4+	AUC (U)	63.24 [58.00–107.9]	82.43 [64.22–255.8]	93.57 [56.89–201.0]	96.21 [87.13–158.10]
	Max (rpv)	1.156 [1.132–1.256]	1.268 [1.174–1.623]	1.321 [1.149–1.477]	1.254 [1.216–1.420]
	*t*_max_ (s)	258.4 [192.1–283.5]	527.5 [327.9 595.3]*	471.1 [288.2–594.3]*	594.7 [290.5–595.5]*
CD8+	AUC (U)	48.76 [38.53–93.22]	88.52 [54.65–327.8]	104.8 [42.14–180.8]	81.55 [58.51–407.1]
	Max (rpv)	1.139 [1.089–1.262]	1.247 [1.124–1.852]	1.318 [1.120–1.434]	1.220 [1.125–1.819]
	*t*_max_ (s)	228.6 [162.1–593.2]	594.3 [294.6–595.6]*	594.4 [422.9–596.1]*	282.7 [232.9–531.0]

Mitochondrial Ca^2+^					

CD4+	AUC (U)	61.64 [37.97–94.43]	66.00 [41.55–95.52]	64.87 [42.90–105.4]	67.60 [49.29–104.2]
	Max (rpv)	1.194 [1.121–1.272]	1.207 [1.103–1.241]	1.149 [1.114–1.281]	1.172 [1.159–1.266]
	*t*_max_ (s)	594.0 [211.9–594.9]	593.3 [384.7–595.3]	595.2 [593.0–596.2]	595.1 [386.6–596.4]
CD8+	AUC (U)	106.9 [71.64–144.5]	87.79 [62.47–101.4]	71.31 [53.46–175.0]	118.8 [67.55–249.9]
	Max (rpv)	1.250 [1.152–1.404]	1.187 [1.130–1.217]	1.154 [1.131–1.400]	1.259 [1.143–1.539]
	*t*_max_ (s)	144.2 [121.6–160.6]	577.1 [167.8–594.2]*	468.8 [165.3–594.7]*	198.7 [169.8–248.4]

Nitric oxide production					

CD4+	AUC (U)	3.318 [−8.315–18.84]	47.76 [1.727–97.18]*	22.20 [−8.903–37.95]	−3.055 [−13.24–9.005]^#^
	Max (rpv)	1.013 [1.000–1.043]	1.113 [1.011–1.282]*	1.046 [1.000–1.081]	1.001 [1.000–1.034]^#^
	*t*_max_ (s)	198.7 [34.55–321.8]	594.3 [228.2–594.8]*	331.2 [0.000–556.5]	107.9 [0.000–252.5]^#^
CD8+	AUC (U)	−9.481 [−19.97–24.30]	41.31 [6.068–109.8]*	9.641 [−11.20–29.92]	−11.80 [−29.93–4.094]^#^
	Max (rpv)	1.003 [1.000–1.051]	1.099 [1.011–1.330]*	1.025 [1.001–1.078]	1.000 [1.000–1.001]^#^
	*t*_max_ (s)	51.08 [0.000–185.2]	594.2 [185.2–594.8]*	289.1 [54.03–553.5]	0.001 [0.000–130.5]^#^

Superoxide generation					

CD4+	AUC (U)	79.27 [64.68–88.90]	75.65 [70.70–92.61]	82.64 [58.40–96.68]	70.99 [56.09–83.99]
CD8+	AUC (U)	73.15 [62.21–80.33]	74.97 [61.28–98.75]	83.87 [58.13–90.74]	60.34 [51.38–85.42]

Data are expressed as median [interquartile range] *versus control *P* < 0.05; ^#^versus before IFX therapy *P* < 0.05; AUC: area under the curve, Max: maximum value, *t*_max _: time to reach maximum.
